# Generalized Mean Fields for Trapped Atomic Bose-Einstein Condensates

**DOI:** 10.6028/jres.101.048

**Published:** 1996

**Authors:** N. P. Proukakis, K. Burnett

**Affiliations:** Clarendon Laboratory, Department of Physics, University of Oxford, Parks Road, Oxford OX1 3PU, United Kingdom; Clarendon Laboratory, Department of Physics, University of Oxford, Parks Road, Oxford OX1 3PU, United Kingdom; National Institute of Standarads and Technology, Gaithersburg, MD 20899-0001 U.S.A.

**Keywords:** evaporative cooling, Hartree-Fock-Bogoliubov approximation, kinetics of Bose-Einstein condensation, mean-field theory, nonlinear Schrödinger equation

## Abstract

We describe generalized time-dependent mean-field equations for partially condensed samples of trapped and evaporatively cooled atoms. These equations give a way of investigating the various order parameters that may be present as well as the existence of a mean value of the field due to condensed atoms. Our approach provides us with a closed system of self-consistent equations for the order parameters present. The equations we derive are shown to reduce to other treatments in the literature in various limits. We also show how the equation of motion method allows us to construct a formalism that can handle the evolution of these mean fields due to two-loop kinetics.

## 1. Introduction to Generalized Mean Fields

A great deal of interest has been generated in Bose-Einstein Condensation (BEC) by the recent experimental observations of this phenomenon in evaporatively cooled alkali gases [1], [2], [3]. These experiments have, in particular, given great impetus to the study of inhomogeneous Bose gases. There are, of course, a fair number of treatments of the Bose gas in the literature, which make use of the various tools of the many body trade and a thorough account of the special features of the physics of homogeneous Bose-condensed liquids is given in [4]. Most treatments deal with Bose-condensed systems that are static and either in, or close to, thermal equilibrium. The majority of studies have also, until recently, been restricted to homogeneous gases, with the notable exceptions of the work of Pitaevskii [5] and Fetter [6]. However, due to the fact that BEC has been experimentally achieved in a trap, there are several more recent studies that have addressed the inhomogeneous case. In this paper we shall present an equation of motion approach [7] that is well-suited to handling both time-dependent and non-equilibrium cases for a trapped (i.e., inhomogeneous) gas. We shall also show how this approach reduces to the conventional *T* ≠ 0 theory in the appropriate limits.

At non-zero temperatures the atomic assembly does, of course, consist of excitations above the condensate which have to be taken properly into account. Perhaps the best known way to do this is based on the finite-temperature Hartree-Fock-Bogoliubov (HFB) equations [8]–[11]. These equations include the effects of the mean value of the atomic field, the effects of thermally excited atoms, as well as the so-called anomalous averages of atomic operators [12]. These anomalous averages represent strong pair correlations between the particles, that can arise when the effective interactions are attractive. The HFB equations can be derived in a variety of ways, e.g., via Green functions, variational principles [8], [11] and through an equation of motion approach. An important discussion of the HFB theory has been recently given by Griffin [13], who describes how the behavior of an inhomogeneous Bose-condensed gas can be obtained for the full temperature range within the context of the Popov approximation [14] (explained below).

In this paper we present what could be considered a generalization of his approach to include the possibility of yet further order parameters of the type discussed by Lalöe [15]. In doing this, we also lay the ground for a fully non-equilibrium technique. We have chosen to work with the equation of motion method, as opposed to other approaches like the non-equilibrium Green function technique (Keldysh Method) [16], [17], as it is somewhat easier to produce kinetic equations and also to give a link to simpler equilibrium theories. Furthermore, the equation of motion approach makes it easier to look at “one-loop” effects away from equilibrium with arbitrary initial conditions. We emphasize that our techniques are aimed at the dilute gases that are being produced by the evaporative cooling method [18]. For those, a kinetic equation approach should be a very accurate and convenient technique. It is also possible to set up efficient simulation techniques based on Monte-Carlo wavefunction methods for a generalized density matrix approach.

We still need, however, to construct a proper self-consistent starting point for the kinetics and employ a consistent decoupling approximation. We believe this to be equivalent to the approach advocated in [19] for the calculation of the time variation of the superconducting order parameter in a Fermi liquid. Central to such an approach lies the idea that the main effects of the excitations can be described through the mean fields (“one-loop” diagrams), whereas all higher order correlation effects can be summed up into kinetic terms terms (i.e., “two-loop” effects). We shall not be able to give a full account of the kinetic terms here, but we will show clearly how they enter our formulation and how they can be computed. The full details of the kinetic terms will be given elsewhere [20].

We start our analysis by considering a group of atoms trapped by an external field. The spatial and temporal behavior can both be fully described, in second quantization, in terms of the boson field operator $\hat{\Psi }(r,\;t)$. We will first outline our approach in terms of $\hat{\Psi }(r,\;t)$, before giving our results for the occupation number representation in the subsequent sections, the latter treatment being better suited to a computational approach to the problem. For the dilute gases under consideration, we assume that the dominant interatomic interactions occur pairwise. The hamiltonian of the system can be thus written in the form

$$\begin{array}{l} \hat{H}=\int {\hat{\Psi }}^{†}(r,\;t)\left[-\frac{\hbar^{2}\nabla_{r}^{2}}{2m}+V_{trap}(r,\;t)\right]\hat{\Psi }(r,\;t)dr\\ +\frac{1}{2}\iint {\hat{\Psi }}^{†}(r,\;t){\hat{\Psi }}^{†}(r^{\prime },\;t)\hat{V}(r-r^{\prime })\hat{\Psi }(r^{\prime },\;t)\hat{\Psi }(r,\;t)drdr^{\prime }. \end{array}$$ (1)

Here $\hat{V}(r-r^{\prime })$ is the interaction between the particles, which, for the dilute bose gas, is conventionally taken to be

$$\hat{V}(r-r^{\prime })=U_{0}\delta (r-r^{\prime }).$$ (2)

Here *U*_0_ is related to the scattering length *a* via $U_{0}=\frac{4\pi \hbar^{2}a}{m}$ [21]. The potential problem of applying this in a self-consistent approach in a 3-dimensional gas is described by Huang et al. [22]. In fact our approach does not rely on this approximation, as will become clear in Sec. 2. For the point of discussion, let us however assume, for the moment, that it is valid; this will enable us to give our initial discussion in terms that link easily with previous accounts.

The operators $\hat{\Psi }(r,\;t)$ satisfy the equal-time boson commutation relations

$$\text{[}\hat{\Psi }(r,\;t),\;\;\hat{\Psi }(r^{\prime },\;t)]=[{\hat{\Psi }}^{†}(r,\;t),\;\;{\hat{\Psi }}^{†}(r^{\prime },\;t)]=0$$ (3)

$$\text{[}\hat{\Psi }(r,\;t),\;\;{\hat{\Psi }}^{†}(r^{\prime },\;t)]=\delta (r-r^{\prime }).$$ (4)

The Heisenberg equation of motion for $\hat{\Psi }(r,\;t)$ then becomes

$$\begin{array}{c} i\hbar \frac{\partial \hat{\Psi }(r,\;t)}{\partial t}=[\left(-\frac{\hbar^{2}\nabla_{r}^{2}}{2m}+V_{\text{trap}}(r)\right)\\ \;+NU_{0}{\hat{\Psi }}^{†}(r,\;t)\hat{\Psi }(r,\;t)]\;\hat{\Psi }(r,\;t). \end{array}$$ (5)

This exact operator equation is the starting point for most quantum-mechanical treatments of a Bose system. We are, in the main, interested in investigating the temporal variation of the coherent (mean-field) relative to the incoherent parts of the boson field operator. We thus follow the usual route of decomposing $\hat{\Psi }(r,\;t)$ as [6]

$$\hat{\Psi }(r,\;t)=\Phi (r,\;t)+\hat{\delta }(r,\;t).$$ (6)

Here $\Phi (r,t)=\langle \hat{\Psi }(r,\;t)\rangle$ expresses the non-vanishing macroscopic value of the Bose field associated with broken symmetry due to the presence of BEC. We note that there are some subtle extra issues that arise in this procedure in the case of small trapped assemblies of atoms. For the point of view of most of this article we shall assume that this is a well-defined quantity. The reader should however bear in mind that in small systems ***Φ*** (***r***, *t*) may evolve in amplitude and phase [23], [24]. In this paper we shall develop a method for determining this evolution.

Using this decomposition we note that we can re-express the interaction term ${\hat{\Psi }}^{†}\hat{\Psi }\hat{\Psi }$ of Eq. (5) in the form

$${\hat{\Psi }}^{†}\hat{\Psi }\hat{\Psi }=|\Phi {|}^{2}\Phi +2|\Phi {|}^{2}\hat{\delta }+2\Phi {\hat{\delta }}^{†}\hat{\delta }+\Phi^{2}{\hat{\delta }}^{†}+\Phi^{*}\hat{\delta }\hat{\delta }+{\hat{\delta }}^{†}\hat{\delta }\hat{\delta }.$$ (7)

The first term in the above expression corresponds to the mean field term which, if it were the only term retained, would convert the mean value of Eq. (5) into the usual Nonlinear Schrödinger Equation (NLSE) or Gross-Pitaevskii equation [25]

$$\begin{array}{c} i\hbar \frac{\partial \Phi (r,\;t)}{\partial t}=\left(-\frac{\hbar^{2}\nabla_{r}^{2}}{2m}+V_{\text{trap}}(r)\right)\Phi (r,\;t)\\ +NU_{0}|\Phi (r,\;t){|}^{2}\Phi (r,\;t). \end{array}$$ (8)

This is the simplest mean field equation describing the atomic assembly and is only strictly valid when all the atoms are condensed, e.g., at temperatures close to zero. The NLSE has already been studied in some detail along with the link to the *T* = 0 elementary excitations (phonons) in isotropic [26] – [29] and anisotropic [30], [31] traps.

The next four terms appearing on the right hand side of Eq. (7) represent the interaction between condensate and excited states. Let us now discuss their meaning. Consider substituting those terms into the operator equation of motion Eq. (5) and taking the average of that expression. The first two terms in the resulting mean-field equation correspond to the direct and exchange Hartree-Fock (HF) terms. The next two terms represent the Bogoliubov correction. In the Popov approximation [13], [14] of the HFB equations, the anomalous averages of the form $\langle \hat{\delta }\;(r,t)\hat{\delta }(r,t)\rangle$ are assumed to be negligible, so that the second of the Bogoliubov corrections is dropped. This approximation can be shown to give accurate results for a homogeneous gas with purely repulsive interactions.

Let us now substitute Eq. (7) in full (i.e., including the $\langle \;{\hat{\delta }}^{†}\hat{\delta }\hat{\delta }\;\rangle$ term) into the operator Eq. (5) and consider its mean value. This gives the exact equation of motion

$$\begin{array}{c} i\hbar \frac{\partial \Phi (r,\;t)}{\partial t}=\left(-\frac{\hbar^{2}\nabla_{r}^{2}}{2m}+V_{\text{trap}}(r)\right)\Phi (r,t)\\ +NU_{0}|\Phi (r,\;t){|}^{2}\Phi (r,\;t)\\ +NU_{0}\{2\Phi (r,\;t)\langle {\hat{\delta }}^{†}(r,\;t){\hat{\delta }}^{†}(r,\;t)\rangle \\ +\Phi^{*}(r,\;t)\langle {\hat{\delta }}^{†}(r,\;t){\hat{\delta }}^{†}(r,\;t)\rangle \}\\ +NU_{0}\langle {\hat{\delta }}^{†}(r,\;t)\hat{\delta }(r,\;t)\hat{\delta }(r,\;t)\rangle . \end{array}$$ (9)

Conventionally, the terms $\langle {\hat{\delta }}^{†}\hat{\delta }\hat{\delta }\;\rangle$ are handled in the self-consistent approximation [13]

$$\begin{array}{l} {\hat{\delta }}^{†}(r,\;t)\hat{\delta }(r,\;t)\hat{\delta }(r,\;t)≃2\langle {\hat{\delta }}^{†}(r,\;t)\hat{\delta }(r,\;t)\rangle \hat{\delta }(r,\;t)\\ +\langle \hat{\delta }(r,\;t)\hat{\delta }(r,\;t)\rangle {\hat{\delta }}^{†}(r,\;t). \end{array}$$ (10)

This implies that such terms vanish trivially in the finite temperature generalization of the NLSE, given by Eq. (9). However, the terms $\langle {\hat{\delta }}^{†}\hat{\delta }\hat{\delta }\;\rangle$ contain the effects of more complex correlations between the particles, which are neglected in the HFB approximation. We have chosen to analyze the effect of these correlations, thus extending the pure mean-field treatments (e.g., Ref. [13]). Such correlations of three particle operators are transiently produced during collisions and thus generate the collisional feed term for the condensate, as discussed in [32], [33]. For the dilute gas, these kinetic effects can be dealt with, as discussed below and in [20]. We note once again that we have chosen to do the calculation in this way, because such treatment gives a full equation of motion that is valid outside thermal equilibrium and can handle essentially arbitrary initial states. The Keldysh non-equilibrium approach [16], [17] should clearly produce results entirely equivalent to ours in the dilute gas limit. One should however bear in mind that the results from two such different formalisms may be difficult to reconcile at first sight.

Let us consider the behavior of triple product averages over time scales that are long compared to the duration of a typical binary atomic collision. A self-consistent evolution may then produce non-vanishing mean values of the triples, which would mean they have to be considered on an equal footing with the other order parameters of the system (i.e., BEC or pairing). Such a possibility has already been discussed in [15], where a cluster expansion approach is used, instead of the usual perturbative expansion in the interatomic potential, for the description of dilute degenerate gases with short-range repulsive interactions. In this paper we derive a self-consistent set of equations that allows for the inclusion of possible non-vanishing triple product mean values, which we shall henceforth refer to as “triplets” in the spirit of Lalöe [15]. Whether such an order parameter can be present in a real system and how it might affect the behavior of the system will be the subject for future study.

We shall therefore procede in a generalized mean-field approach for which triplets are retained as a possibility. We are particularly interested in laying the ground for the study of such self-consistent solutions and their role in the “macroscopic” behavior of a gas. When looking at the structure of Eq. (9) it is clear that in order to obtain a closed set of equations for the various possible order parameters, we shall need the evolution of the averages of products of two and three fluctuation operators δ^(†)^(***r***, *t*). In our self-consistent coupled equations, all operators being averaged over have the same time label which we shall not, for the sake of convenience, give explicitly in the equations. In expressing the equations for such averages we have used suffixes 1 and 2 to indicate whether the boson field operators are acting on spatial coordinates ***r*** or ***r***′ respectively. For the binary products, corresponding to what we shall term the generalized density matrix, we find the following equations of motion

$$\begin{array}{l} i\hbar \frac{\partial }{\partial t}\langle {\hat{\delta }}_{1}^{†}{\hat{\delta }}_{2}\rangle =\left[-\frac{\hbar^{2}}{2m}(\nabla_{2}^{2}-\nabla_{1}^{2})+(V_{\text{trap}}^{\left(2\right)}-V_{\text{trap}}^{\left(1\right)})\right]\langle {\hat{\delta }}_{1}^{†}{\hat{\delta }}_{2}\rangle \\ \;+NU_{0}\{\Phi_{2}^{2}\langle {\hat{\delta }}_{1}^{†}{\hat{\delta }}_{2}^{†}\rangle +2|\Phi_{2}{|}^{2}\langle {\hat{\delta }}_{1}^{†}{\hat{\delta }}_{2}\rangle +\langle {\hat{\delta }}_{1}^{†}{\hat{\delta }}_{2}^{†}{\hat{\delta }}_{2}{\hat{\delta }}_{2}\rangle \}\\ \;-NU_{0}\left[2\Phi_{2}\langle {\hat{\delta }}_{1}^{†}{\hat{\delta }}_{2}^{†}{\hat{\delta }}_{2}\rangle +\Phi_{2}^{*}\langle {\hat{\delta }}_{1}^{†}{\hat{\delta }}_{2}{\hat{\delta }}_{2}\rangle \right]\\ \;+NU_{0}\{(\Phi_{1}^{*}{)}^{2}\langle {\hat{\delta }}_{1}{\hat{\delta }}_{2}\rangle +2|\Phi_{1}{|}^{2}\langle {\hat{\delta }}_{1}^{†}{\hat{\delta }}_{2}\rangle \\ \;-\langle {\hat{\delta }}_{1}^{†}{\hat{\delta }}_{1}^{†}{\hat{\delta }}_{1}{\hat{\delta }}_{2}\rangle \}\\ \;+NU_{0}\left[2\Phi_{1}^{*}\langle {\hat{\delta }}_{1}^{†}{\hat{\delta }}_{1}{\hat{\delta }}_{2}\rangle +\Phi_{1}\langle {\hat{\delta }}_{1}^{†}{\hat{\delta }}_{1}^{†}{\hat{\delta }}_{2}\rangle \right] \end{array}$$ (11)

$$\begin{array}{l} i\hbar \frac{\partial }{\partial t}\langle {\hat{\delta }}_{1}{\hat{\delta }}_{2}\rangle =\left[-\frac{\hbar^{2}}{2m}(\nabla_{1}^{2}+\nabla_{2}^{2})+(V_{\text{trap}}^{\left(1\right)}-V_{\text{trap}}^{\left(2\right)})\right]\langle {\hat{\delta }}_{1}{\hat{\delta }}_{2}\rangle \\ \;+NU_{0}\{\Phi_{2}^{2}\langle {\hat{\delta }}_{2}^{†}{\hat{\delta }}_{1}^{†}\rangle +2|\Phi_{2}{|}^{2}\langle {\hat{\delta }}_{1}{\hat{\delta }}_{2}\rangle +\langle {\hat{\delta }}_{2}^{†}{\hat{\delta }}_{1}{\hat{\delta }}_{2}{\hat{\delta }}_{2}\rangle \}\\ \;+NU_{0}\left[2\Phi_{2}\langle {\hat{\delta }}_{1}{\hat{\delta }}_{2}^{†}{\hat{\delta }}_{2}\rangle +\Phi_{2}^{*}\langle {\hat{\delta }}_{1}{\hat{\delta }}_{2}{\hat{\delta }}_{2}\rangle \right]\\ \;+\delta_{12}^{k}(\Phi_{2}^{2}+\langle {\hat{\delta }}_{2}{\hat{\delta }}_{2}\rangle )\\ \;+NU_{0}\{\Phi_{1}^{2}\langle {\hat{\delta }}_{1}^{†}{\hat{\delta }}_{2}\rangle +2|\Phi_{1}{|}^{2}\langle {\hat{\delta }}_{1}{\hat{\delta }}_{2}\rangle \\ \;+\langle {\hat{\delta }}_{1}^{†}{\hat{\delta }}_{1}{\hat{\delta }}_{1}{\hat{\delta }}_{2}\rangle \}\\ \;+NU_{0}\left[2\Phi_{1}\langle {\hat{\delta }}_{1}^{†}{\hat{\delta }}_{1}{\hat{\delta }}_{2}\rangle +\Phi_{1}^{*}\langle {\hat{\delta }}_{1}{\hat{\delta }}_{1}{\hat{\delta }}_{2}\rangle \right], \end{array}$$ (12)

here $\delta_{12}^{k}$ denotes a Kronecker delta.

These equations are again exact, although fairly useless without any approximations. We note that, apart from averages of products of three fluctuation operators, terms with products of four such operators appear on the right-hand side of the above equations. These represent more complex correlations between particles undergoing collisions and we will make appropriate approximations for them as we proceed.

In principle, one could work out the equations for the rate of change of both these quantities which would in turn depend on higher order correlations. It is, however, essential to terminate this coupling to higher and higher order parameters by making some suitable truncation, which is a very natural procedure in a weakly-coupled gas [7]. In this paper, we have chosen to include the possibility of mean values of triple products in our model and reduce higher particle correlations to lower ones (via an approximate version of Wick’s theorem). By including all correlations up to the triplets, we can describe the effects of the kinetics on the evolution of the condensate in a fully self-consistent mean-field approach. In order to obtain the desired closed set of coupled equations within this framework, we need of course to consider the equations of motion for averages of products of three fluctuation operators, which is done in Sec. 4.

The method described in this paper can, in principle, be used for calculating the relative importance of these various order parameters. The possible appearance of a pairing or other order parameters in the case of attractive interactions has been anticipated for some time [34]. In [35] Stoof has discussed the possibility of the anomalous average $\langle \hat{\delta }\hat{\delta }\rangle$ becoming the important order parameter in the case of a homogeneous gas with attractive interactions. We would like to examine the closely related issue for a trapped inhomogeneous gas.

In the next section we repeat the above analysis in the occupation number formalism and discuss the various approximations made in more detail. We then show how the equations can be cast in a generalized density matrix form (Sec. 3) and derive equations of motion for the triplets (Sec. 4). We further illustrate the consistency of the derived equations with simpler ones found in the literature (Sec. 5) and argue how our analysis can be used to set the basis for a full non-equilibrium theory.

## 2. Mean Field Equations for a Trapped Atomic System

In the occupation number representation the hamiltonian for pairwise interactions has the form

$$\begin{array}{l} \hat{H}=\sum_{rs}\langle r|\hat{\Xi }|s\rangle {\hat{a}}_{r}^{†}(t){\hat{a}}_{s}(t)\\ \;+\frac{1}{2}\sum_{rsmn}\langle rs|\hat{V}|mn\rangle {\hat{a}}_{r}^{†}(t){\hat{a}}_{s}^{†}(t){\hat{a}}_{n}(t){\hat{a}}_{m}(t). \end{array}$$ (13)

In this equation the operator $\hat{\Xi }$ contains the kinetic energy as well as the trap potential and $\langle rs|\hat{V}|mn\rangle$ represents the interaction potential between a pair of particles. This is defined by

$$\begin{array}{l} \langle rs|\hat{V}|mn\rangle =\\ \iint \psi_{r}^{*}(r)\psi_{r}^{*}(r^{\prime })V(r-r^{\prime })\psi_{n}(r)dr\;dr^{\prime }. \end{array}$$ (14)

Here the *ψ_i_* are the spatial parts of the Fock space decomposition of $\hat{\Psi }(r,t)$ given by

$$\hat{\Psi }(r,t)=\sum_{i}\psi_{i}(r){\hat{a}}_{i}(t).$$ (15)

Analogous to Eq. (6) we now decompose the time-dependent single-particle operators according to

$${\hat{a}}_{i}(t)=z_{i}(t)+{\hat{c}}_{i}(t).$$ (16)

This decomposition is extremely useful as it allows the application of Wick’s theorem for decomposing higher order correlations into lower order products.

Initially we consider the temporal dependence of the mean field which yields the exact equation^1^ [analogous to Eq. (9)]

$$\begin{array}{l} i\hbar \frac{dz_{i}(t)}{dt}=\sum_{r}\langle i|\hat{\Xi }|r\rangle z_{r}(t)+\sum_{rms}\langle is|\hat{V}|mr\rangle \\ \;\{2\rho_{ms}(t)z_{r}(t)+z_{s}^{*}(t)z_{m}(t)z_{r}(t)+\kappa_{mr}(t)z_{s}^{*}(t)\\ \;+\langle c_{s}^{†}(t)c_{r}(t)c_{m}(t)\rangle \}. \end{array}$$ (17)

Here we have defined the one-body density matrix ***ρ*** (*t*) and the two-body or pair density matrix ***κ*** (*t*) by their respective matrix elements^2^

$$\rho_{ij}(t)\equiv \langle i|\rho (t)|j\rangle \equiv \langle c_{j}^{†}(t)c_{i}(t)\rangle$$ (18)

$$\kappa_{ij}(t)\equiv \langle ij|\kappa (t)\rangle \equiv \langle c_{j}(t)c_{i}(t)\rangle .$$ (19)

We note that ***ρ*** (*t*) and ***κ*** (*t*) are both *n* × *n* matrices, where *n* is the number of single-particle states.

The equation of motion for the single-particle density matrix ***ρ****_ij_*(*t*) is given by

$$\begin{array}{c} i\hbar \frac{d\rho_{\mathrm{ij}}(t)}{dt}=-\langle [\hat{H},a_{j}^{†}(t)a_{j}(t)]\rangle \\ \;-i\hbar z_{j}^{*}\frac{dz_{i}(t)}{dt}-i\hbar z_{i}\frac{dz_{j}^{*}(t)}{dt} \end{array}$$ (20)

with

$$\begin{array}{l} \left[\hat{H},a_{j}^{†}(t)a_{i}(t)]=\sum_{r}\langle r|\hat{\Xi }|j\rangle a_{r}^{†}(t)a_{i}(t\right)\\ \;-\sum_{r}\langle i|\hat{\Xi }|r\rangle a_{j}^{†}(t)a_{r}(t)\\ \;+\frac{1}{2}\sum_{rsm}\langle rs|\hat{V}|mj\rangle a_{r}^{†}(t)a_{s}^{†}(t)a_{m}(t)a_{i}(t)\\ \;-\frac{1}{2}\sum_{rmn}\langle ir|\hat{V}|mn\rangle a_{j}^{†}(t)a_{r}^{†}(t)a_{n}(t)a_{m}(t). \end{array}$$ (21)

We now proceed to decompose operators *a_i_*^(†)^ (*t*) according to Eq. (16). Hence (upon suppressing temporal dependence) the exact form of Eq. (20) contains terms with the following structure (with the appropriate in-dices):

$$\begin{array}{l} \;\langle \hat{\Xi }\rangle [z^{*}z,\;\langle c^{†}c\rangle ]\\ \;\text{and}\\ \langle \hat{V}\rangle [z^{*}z^{*}zz,\;\langle c^{†}c\rangle z^{*}z,\;\langle cc\rangle z^{*}z^{*},\langle c^{†}cc\rangle z^{*},\;\langle c^{†}c^{†}cc\rangle ] \end{array}$$

and their hermitian conjugate quantities. At this point we will simplify our expressions by assuming that quantities of the form 〈*c*^†^*c*^†^*cc*〉 can be approximated by their mean-field values according to the conventional form of Wick’s theorem

$$\begin{array}{l} \langle c_{r}^{†}c_{s}^{†}c_{m}c_{n}\rangle =\\ \;\langle c_{r}^{†}c_{m}\rangle \langle c_{s}^{†}c_{n}\rangle +\langle c_{r}^{†}c_{n}\rangle \langle c_{s}^{†}c_{m}\rangle +\langle c_{r}^{†}c_{s}^{†}\rangle \langle c_{m}c_{n}\rangle \\ \;=\rho_{mr}\rho_{ns}+\rho_{nr}\rho_{ms}+\kappa_{rs}^{*}\kappa_{mn}. \end{array}$$ (22)

In this approximation, we are essentially assuming that the effects of higher order correlations (such as 〈*c*^†^*c*^†^*c*^†^*cc*〉) on the mean values of 〈*c*^†^*c*^†^*cc*〉 can be neglected. Within our mean-field approximation defined by Eq. (22), we obtain the following equations of motion

$$\begin{array}{l} i\hbar \frac{d\rho_{ij}}{dt}=\sum_{r}[h_{ir}\rho_{rj}-\rho_{ir}h_{rj}]-\sum_{r}[\kappa_{ir}\Delta_{rj}^{*}-\Delta_{ir}\kappa_{rj}^{*}]\\ \;+\sum_{rmn}\langle ir|\hat{V}|mn\rangle \{\langle c_{j}^{†}c_{r}^{†}c_{m}\rangle z_{n}\\ \;+\langle c_{j}^{†}c_{r}^{†}c_{n}\rangle z_{m}+\langle c_{j}^{†}c_{n}c_{m}\rangle z_{r}^{*}\}\\ \;-\sum_{rsm}\langle rs|\hat{V}|mj\rangle \{\langle c_{r}^{†}c_{m}c_{i}\rangle z_{s}^{*}\\ \;+\langle c_{s}^{†}c_{m}c_{i}\rangle z_{r}^{*}+\langle c_{r}^{†}c_{s}^{†}c_{i}\rangle z_{m}\}. \end{array}$$ (23)

Here we have defined the time-dependent Hartree-Fock hamiltonian $\hat{h}(t)$ and the pairing field $\hat{\Delta }(t)$ by their respective matrix elements [11] as follows:

$$h_{ij}\equiv \langle i|\hat{h}|j\rangle \equiv \langle i|\hat{\Xi }|j\rangle +2\sum_{kl}\langle ik|\hat{V}|lj\rangle [z_{k}^{*}z_{l}+\rho_{lk}]$$ (24)

$$\Delta_{ij}\equiv \langle ij|\hat{\Delta }\rangle \equiv \sum_{kl}\langle ij|\hat{V}|kl\rangle [\kappa_{kl}+z_{k}z_{l}].$$ (25)

We note that in an alternative (variational) approach [11] (see Appendix A), these may be defined as

$$h_{ij}(t)\equiv \frac{\partial \hat{E}}{\partial \rho_{ji}(t)}\;\Delta_{ij}(t)\equiv \frac{\partial \hat{E}}{\partial \kappa_{ji}^{*}(t)}$$ (26)

where Ê=Ê[z,ρ,κ] is the energy functional of the system.

The equations of motion for the pair matrix ***κ*** can be derived along the same lines as above. Consideration of the term 〈[*Ĥ*, *a_j_* (*t*)*a_i_* (*t*)]〉 gives rise to the correlation $\langle a_{j}a_{r}^{†}a_{n}a_{m}\rangle$ (where *r*, *m*, *n* represent indices that are being summed over). By using the decomposition equation [Eq. (16)] for obtaining correlations of fluctuation operators and imposing the boson commutation rules for the *c*-operators, we obtain the term $\langle c_{r}^{†}c_{j}c_{n}c_{m}\rangle$, which is handled in the mean-field approximation in an analogous fashion to Eq. (22).

Hence, the equation analogous to Eq. (12) reads

$$\begin{array}{l} i\hbar \frac{d\kappa_{ij}}{dt}=\sum_{r}[h_{ir}\kappa_{rj}+\kappa_{ir}h_{rj}^{*}]+\sum_{r}[\rho_{ir}\Delta_{rj}+\Delta_{ir}\rho_{rj}^{*}]+\Delta_{ij}\\ \;+\sum_{rmn}\langle ir|\hat{V}|mn\rangle \{\langle c_{r}^{†}c_{j}c_{n}\rangle z_{m}\\ \;+\langle c_{r}^{†}c_{j}c_{m}\rangle z_{n}+\langle c_{j}c_{n}c_{m}\rangle z_{r}^{*}\}\\ \;+\sum_{smn}\langle js|\hat{V}|mn\rangle \{\langle c_{s}^{†}c_{n}c_{i}\rangle z_{m}\\ \;+\langle c_{s}^{†}c_{m}c_{i}\rangle z_{n}+\langle c_{n}c_{m}c_{i}\rangle z_{s}^{*}\}. \end{array}$$ (27)

The parameters *z*, ***ρ***, ***κ*** and 〈*c*^(†)^*cc*〉 can be treated (self-consistently) as suitable order parameters for the system, provided they are well-defined. This could be true either in a completely static or a slowly-varying situation. It is however also possible that the kinetic correlation terms 〈*c*^(†)^*cc*〉 are established on time-scales much shorter than the mean evolution times of the parameters *z*, ***ρ***, and ***κ***. This is equivalent to saying that the collision duration relevant to the evolution of 〈*c*^(†)^*cc*〉 must be much smaller than the mean time between collisions. This should hold for dilute weakly-interacting Bose gases for which *n*_t_a^3^ ≪ 1 where *n*_t_ is the number density in the trap.

In the next section we write these equations in a more compact generalized matrix form. We then derive equations of motion for the triplets (Sec. 4) and show that if the latter are neglected (Sec. 5), the equations derived in this paper are consistent with simplified versions appearing in the literature.

## 3. The Equations of Motion in a Generalized Density Matrix Form

Equations (23) and (27) can be cast in a generalized matrix form

$$i\hbar \frac{d\rho }{dt}=[h,\rho ]-(\kappa \Delta^{*}-\Delta \kappa^{*})+(M-{\tilde{M}}^{*})$$ (28)

$$i\hbar \frac{d\kappa }{dt}=[h\kappa +\kappa h^{*}]+(\rho \Delta +\Delta \rho^{*})+\Delta +(N+\tilde{N})$$ (29)

where the matrices *M* and *N* have been defined in terms of their elements

$$\begin{array}{l} M_{ij}=\sum_{rms}\langle is|\hat{V}|mr\rangle [\langle c_{j}^{†}c_{s}^{†}c_{m}\rangle z_{r}\\ \;+\langle c_{j}^{†}c_{s}^{†}c_{r}\rangle z_{m}+\langle c_{j}^{†}c_{r}c_{m}\rangle z_{s}^{*}] \end{array}$$ (30)

$$\begin{array}{l} N_{ij}=\sum_{rms}\langle is|\hat{V}|mr\rangle [\langle c_{s}^{†}c_{m}c_{j}\rangle z_{r}\\ \;+\langle c_{j}^{†}c_{r}c_{j}\rangle z_{m}+\langle c_{r}c_{m}c_{j}\rangle z_{s}^{*}] \end{array}$$ (31)

and $\tilde{N}$ represents the transpose of matrix *N.*

It is straightforward to show that these can be written in matrix notation as

$$i\hbar \frac{dR}{dt}=\eta [H,R]+K$$ (32)

where all matrices are of the form 2*n*×2*n*. Here we have defined the generalized boson density matrix *R* by

$$R=\left(\begin{array}{cc} \rho & \kappa \\ -\kappa^{*} & -(\rho^{*}+11) \end{array}\right)$$ (33)

and the matrix *η* by

$$\eta =\left(\begin{array}{cc} 11 & 0\\ 0 & -11 \end{array}\right)$$ (34)

where *11* is the unity matrix of the *n*-dimensional Fock space.

For convenience, we have also defined the boson quasiparticle hamiltonian

$$H=\left(\begin{array}{cc} h & -\Delta \\ \Delta^{*} & -h \end{array}\right)$$ (35)

We note that the matrices *R* and *H* are not the same as those defined by Blaizot and Ripka [11] as discussed in Appendix A.

We have further defined the matrix

$$K=\left(\begin{array}{cc} M-{\tilde{M}}^{*} & \left(N+\tilde{N}\right)\\ -{\left(N+\tilde{N}\right)}^{*} & -{\left(M-{\tilde{M}}^{*}\right)}^{*} \end{array}\right)$$ (36)

which gives the effect of the triplets on the single-particle and pair excitations.

Thus far, we have derived equations of motions for ***ρ*** and ***κ*** which involve triplets. In order to get a closed set of mean values, we need the equations of motion for the triplets which we derive below.

## 4. Equations of Motion for the Triplets

Before deriving the equations of motion for triplets of the form 〈*c*^(†)^*cc*〉 we would like to point out that they can be used in two ways. Firstly, they allow us to construct a self-consistent generalized mean-field theory. Furthermore, they offer the possibility of including kinetic effects of condensate formation.

We define the time-dependent *n*×*n*×*n* rank 3 triplet tensors $\tilde{\gamma }(t)$ and $\tilde{\lambda }(t)$ by their respective elements

$$\gamma_{ijk}=\langle c_{i}c_{j}c_{k}\rangle$$ (37)

and

$$\lambda_{ijk}=\langle c_{i}^{†}c_{j}c_{k}\rangle$$ (38)

The equations of motion for the triplets can be obtained by following the procedure of Sec. 2. We note that this leads to very complex expressions, so the account will be kept very brief here.

Consider initially the equation of motion fot the triplet *γ_ijk_*. Upon imposing the decomposition equation [Eq. (16)], we note that the right hand side of the resulting equation contains—apart from the simpler correlations—correlations of four and five single-particle fluctuation operators. We assume that the effects of correlations of six or more operators (present on the right hand side of higher equations of motion) are negligible when looking at the behavior of the system during a typical collision. This means that we set all terms 〈*cccccc*〉 ≃ (〈*cc*〉)^3^, (〈*ccc*〉)^2^ (appearing in the equations of motion) to zero, irrespective of their indices or whether they contain products of normal or anomalous averages. Correlations of four fluctuation operators are handled in the mean-field approximation Eq. (22). In treating the equation of motion for $\tilde{\gamma }$ we also use Wick’s theorem to decompose correlations of five fluctuation operators in the form

$$\begin{array}{l} \langle c_{s}^{†}c_{i}c_{k}c_{n}c_{m}\rangle =\rho_{is}\gamma_{knm}+\rho_{ks}\gamma_{inm}+\rho_{ns}\gamma_{ikm}+\rho_{ms}\gamma_{ikn}\\ \;+\kappa_{ki}\lambda_{snm}+\kappa_{ni}\lambda_{skm}+\kappa_{mi}\lambda_{skn}+\kappa_{nk}\lambda_{sim}\\ \;+\kappa_{mk}\lambda_{sin}++\kappa_{mn}\lambda_{sik}. \end{array}$$ (39)

It is essential that the above decomposition is in accord with the mean field approximation defined by Eq. (22). Strictly speaking Eq. (22) should contain an extra term on the right hand side due to the effects of five particle operator correlations, and these would have to be included in a treatment that went beyond the mean-field approach.

Writing the resulting equation of motion in more compact notation we obtain

$$\begin{array}{l} i\hbar \frac{d}{dt}(\gamma_{ijk})=\{h,\gamma {\}}_{ijk}\{\Delta ,\lambda {\}}_{ijk}+(W_{ijk}+W_{jki}+W_{kij})\\ \;+(\Gamma_{ijk}+\Gamma_{jki}+\Gamma_{kij}) \end{array}$$ (40)

where we have defined the following quantities

$$\begin{array}{l} \{h,\gamma {\}}_{ijk}=(h\gamma {)}_{ijk}+(h\gamma {)}_{jki}+(h\gamma {)}_{kij}\\ \;=\sum_{r}\left(h_{ir}\gamma_{rjk}+h_{jr}\gamma_{rki}+h_{kr}\gamma_{rij}\right) \end{array}$$ (41)

$$\begin{array}{l} \{\Delta ,\lambda {\}}_{ijk}=(\Delta \lambda {)}_{ijk}+(\Delta \lambda {)}_{jki}+(\Delta \lambda {)}_{kij}\\ \;=\sum_{r}\left(\Delta_{ir}\lambda_{rjk}+\Delta_{jr}\lambda_{rki}+\Delta_{kr}\lambda_{rij}\right) \end{array}$$ (42)

$$\begin{array}{l} W_{ijk}=\sum_{rms}\langle is|\hat{V}|mr\rangle [\kappa_{mj}L_{skr}+\kappa_{mk}L_{sjr}\\ \;+\rho_{js}\gamma_{krm}+\rho_{ks}\gamma_{jrm}] \end{array}$$ (43)

$$L_{rms}=2\rho_{rm}^{*}z_{s}+z_{r}^{*}\kappa_{ms}+2\lambda_{rms}$$ (44)

$$\Gamma_{ijk}=\sum_{ms}\langle ij|\hat{V}|ms\rangle [2\kappa_{km}z_{s}+\gamma_{kms}].$$ (45)

Similarly we have

$$\begin{array}{l} i\hbar \frac{d}{dt}(\lambda_{ijk})=(\lambda h^{*}{)}_{ijk}+(\lambda h^{*}{)}_{ikj}-(h^{*}\lambda {)}_{ijk}\\ \;+(\lambda^{*}\Delta {)}_{jik}+(\lambda^{*}\Delta {)}_{kij}-(\Delta^{*}\gamma {)}_{ijk}\\ \;+(X_{kji}+X_{jki}+Y_{ijk}^{*})+\Lambda_{jki} \end{array}$$ (46)

$$\begin{array}{l} X_{kji}=\sum_{rms}\langle ks|\hat{V}|mr\rangle [\kappa_{mj}J_{ris}+\rho_{ri}L_{sjm}\\ \;+\kappa_{is}^{*}\gamma_{rmj}+\rho_{js}\lambda_{mr}] \end{array}$$ (47)

$$\begin{array}{l} Y_{ijk}=\sum_{rms}\langle is|\hat{V}|mr\rangle [\rho_{mj}J_{rks}+\rho_{mk}J_{rjs}\\ \;+\kappa_{sj}^{*}\lambda_{kmr}+\kappa_{sk}^{*}\lambda_{jmr}] \end{array}$$ (48)

$$J_{rms}=2z_{r}\kappa_{ms}^{*}+\rho_{rm}z_{s}^{*}+2\lambda_{rms}^{*}$$ (49)

$$\Lambda_{jki}=\sum_{ms}\langle jk|\hat{V}|ms\rangle [2\rho_{mi}z_{s}+\lambda_{ims}].$$ (50)

There are a range of ways in which we can make use of these equations. Firstly, we could solve Eqs. (17), (23), (27), (40), and (46) self-consistently as a generalization of the HFB equations that includes the extra anomalous averages. Exploring the phase diagram for the various order parameters will, however, in itself be an enormous task. It will, nonetheless, be an essential one in the study of these new evaporatively cooled assemblies. In the first instance an examination of the HFB-Popov behaviour will be an important advance. The next step should be the study of how 〈*cc*〉 and 〈*c*^(†)^*cc*〉 arise in the presence of attractive interactions. We believe that this will be an important theme in the next few years. We can also use these equations to investigate the damping of time dependent solutions that come from one-loop processes, e.g., the Landau damping discussed by Payne and Griffin [36] for the homogenous case. This means we can explore the range of validity of the simpler NLSE approaches to condensate time evolution.

These equations can also be used to treat condensation kinetics. It should however be pointed out that a completely general treatment that also includes kinetics of excited states, further requires us to look at the equations of motion of terms 〈*c*^(†)^*c* ^(†)^*cc*〉, where the algebraic complexity becomes quite formidable.

## 5. Reduction to Simpler Equations

We now neglect the triplets and obtain simpler equations in appropriate limits, some of which can be found in the literature.

### 5.1 The Nonlinear Schrödinger Equation

If we further ignore single-particle ***ρ*** and pair ***κ*** excitations in the time-dependent equation for *z_i_* (*t*) [Eq. (17)] and restore explicit time-dependence we end up with the mean-field equation

$$i\hbar \frac{dz_{i}(t)}{dt}=\sum_{r}h_{ir}^{\left(0\right)}(t)z_{r}(t)$$ (51)

where the time-dependent mean-field Hartree-Fock hamiltonian ***h***^(0)^(*t*) is defined by

$$h_{ir}^{\left(0\right)}(t)=\langle i|\hat{\Xi }|r\rangle +\langle is|\hat{V}|mr\rangle z_{s}^{*}(t)z_{m}(t).$$ (52)

One should note that the interaction potential appearing in this equation is the original one. The replacement of this by the two-body *T*-matrix is straightforward but relies on a decoupling—ladder approximation—in the equations of motion. We shall give the explicit account of this in a future publication. With this replacement Eq. (51) becomes the analogue of the NLSE [Eq. (8)].

$$\begin{array}{l} i\hbar \frac{\partial \Phi (r,t)}{\partial t}=\left(-\frac{\hbar^{2}\nabla_{r}^{2}}{2m}+V_{\text{trap}}(r)\right)\\ \;\Phi (r,t)+NU_{0}|\Phi (r,t){|}^{2}\Phi (r,t) \end{array}$$ (53)

in the occupation number representation we note that Eq. (15) can be equivalently written in the form

$$\Phi (r,t)=\sum_{i}\phi_{i}(r)z_{i}(t)$$ (54)

This confirms that we can reconstruct the NLSE by multiplying Eq. (51) by $\frac{\phi_{i}(r)}{\sqrt{N}}$ and summing over all indices *i*. The factor $\sqrt{N}$ has been included so as to re-scale the time-dependent coefficients *z_i_*(*t*) which obey the normalization condition

$$\sum_{i}z_{i}^{*}(t)z_{i}(t)=N.$$ (55)

At *T* = 0 the whole system is condensed in the lowest energy eigenstate and since according to Eq. (54) all the time-dependence has been included in the coefficients *z_i_* (*t*), we can re-express these as

$$z_{i}(t)=z_{i}e^{\frac{i\mu t}{h}}$$ (56)

where the *z_i_* are now simply constant complex numbers and *µ* is the ground state chemical potential. Clearly Eq. (51) then reduces to the time-independent NLSE [11]

$$\sum_{r}h_{ir}^{\left(0\right)}z_{r}=\mu z_{i}.$$ (57)

Here we see that we can trivially transform from the time-dependent to the time-independent picture, merely by setting the time-dependent term on the left-hand side to zero and simultaneously replacing the time-dependent mean-field Hartree-Fock hamiltonian

$$h_{ij}^{\left(0\right)}(t)\equiv \langle i|\hat{\Xi }|j\rangle +\sum_{kl}\langle ik|\hat{V}|lj\rangle z_{k}^{*}(t)z_{l}(t)$$ (58)

by the time-independent one given by

$$h_{ij}^{\left(0\right)}(t)\equiv \langle i|\hat{\Xi }-\mu |j\rangle +\sum_{kl}\langle ik|\hat{V}|lj\rangle z_{k}^{*}z_{l}.$$ (59)

We note that this remains true even at non-zero temperatures, when the second term also contains correlations due to the excited particles.

### 5.2 Static Equations

In this section we will show that Eqs. (23) and (27) are also consistent with the *T* > 0 static HFB equations that we have additionally derived from a variational principle approach in Appendix A. Neglecting all time dependence in Eqs. (28) and (29), we thus obtain the static matrix equations

$$\left[h,\rho ]=(\kappa \Delta^{*}-\Delta \kappa^{*}\right)$$ (60)

$$[h\kappa +\kappa h^{*}]+(\rho \Delta +\Delta \rho^{*})+\Delta =0$$ (61)

However, to solve these equations self-consistently, we also need an equation normalizing the vector ***z***. We use the substitution $z_{i}(t)=z_{i}e^{-\frac{i\mu t}{h}}$ in Eq. (17) to obtain the static equation

$$\begin{array}{l} \sum_{r}\langle i|\hat{\Xi }-\mu |r\rangle z_{r}+\\ \sum_{rms}\langle is|\hat{V}|mr\rangle \{2\rho_{ms}z_{r}+z_{s}^{*}z_{m}z_{r}+\kappa_{mr}z_{s}^{*}\}=0 \end{array}$$ (62)

where the chemical potential *µ* appears as discussed earlier.

This can be re-expressed as

$$\sum_{r}(h_{ir}^{c}z_{r}+\Delta_{ir}^{c}z_{r}^{*})=0$$ (63)

where

$$h_{ij}^{c}\equiv \langle i|\hat{\Xi }-\mu |j\rangle +\sum_{kl}\langle ik|\hat{V}|lj\rangle [z_{k}^{*}z_{l}+2\rho_{lk}]$$ (64)

and

$$\Delta_{ij}^{c}\equiv \sum_{kl}\langle ij|\hat{V}|lkl\rangle \kappa_{kl}.$$ (65)

More generally, we obtain the matrix equations [11]

$$\left(\begin{array}{cc} h^{c} & \Delta^{c}\\ {\left(\Delta^{c}\right)}^{*} & {\left(h^{c}\right)}^{*} \end{array}\right)\left(\begin{array}{c} z\\ z^{*} \end{array}\right)=0$$ (66)

which give us the values of all *z_i_* for fixed values of *h^c^* and *∆^c^*. Equations (60), (61) and (66) constitute the full set of static Hartree-Fock-Bogoliubov equations which can be solved self-consistently. If we set *∆* = ***κ*** = 0, we thus obtain the static self-consistent HFB description of a condensate with elementary excitations in the Popov approximation [14], which further reduces to the time-independent NLSE for ***ρ*** = 0.

## 6. Discussion

In this paper we have developed a time-dependent self-consistent generalized mean-field method for describing a trapped and partially condensed atomic system. Central to our approach lies the operator decomposition equation [Eq. (6)] (or equivalently Eq. (16)) into a mean field and a fluctuating part. This decomposition is reasonable, as we do not make any assumptions about the initial value of the wavefunction *Φ* (***r***, *t*), or its evolution. Consequently, our treatment should remain valid over a wide range of conditions, e.g., for cases close to the transition temperature *T*_c_. Our equations allow for the possibility of other order parameters and give us a way of investigating their possible role in an evaporatively cooled gas.

In our treatment, we have considered an inhomogeneous gas, which implies that the excited states of the system are strongly influenced by the finite size of the trapped assembly. This natural length scale will, of course, lead to the presence of a gap in the excitation spectrum, as opposed to the gapless spectrum of the homogeneous gas.

Let us initially assume that the triplets are small in comparison with the usual HFB order parameters. In the case of moderately-sized condensates, the solutions of the HFB equations will hence be a good basis, as long as the kinetic processes produce collisional widths that are less than the separation of the levels. However, as the number of atoms in the trap is increased, we will approach the so-called quasi-homogeneous limit for the condensate. This happens when the healing length for the gas is much smaller than the size of the condensate. In that regime, it becomes essential to describe the mean fields by means of local densities [i.e., *z*(*r*), ***ρ***(*r*), and ***κ***(*r*)]. We thus obtain locally well-defined quasiparticles (in the sense that their collisional widths are smaller than the characteristic frequencies) in regions specified by the healing length of the atomic assembly under consideration. This approach has been employed by Dorfman and Kirkpatrick to derive transport coefficients for the dilute Bose gas [32].

The discussion above relies on assumption that the HFB states for the trap as a whole constitute the most sensible state. This, in turn, implies that we should, in order to be consistent, assume that any kinetic rates are less than the frequency separation of the HFB excitations, which seems reasonable for the case of condensates with modest numbers of particles. Our equations are, however, not limited to the pure HFB regime. For example, in the case of repulsive interactions, both pairing and triplets may be treated purturbatively and the analysis extended beyond HFB.

## Footnotes

1 Due to the extensive use of the operators ${{\hat{a}}_{i}}^{\left(†\right)}$, ${{\hat{c}}_{i}}^{\left(†\right)}$ we have dropped the explicit operator “hat” notation (^).

2 We would like to point out here that these matrices transform differently under a unitary transformation, which is implicit in the notation 〈*i*|***ρ*** (*t*)|*j*〉 and 〈*ij* |***κ***(*t*)〉. As a matter of fact, ***κ*** (*t*), unlike ***ρ*** (*t*), transforms as a matrix only under real orthogonal transformations.

## 7. Appendix A. Proof of the Static Generalized Equations using a Variational Principle

In this Appendix we derive the static form of the generalized quasiparticle equations of motion using the variational principle approach employed, in detail, by Blaizot and Ripka for fermions [11].

Consider a system in thermal equilibrium described by a statistical density matrix *D* given by

$$D=\frac{e^{-\beta Q}}{Z_{0}}$$ (67)

where *Z*_0_ is given by

$$Z_{0}=Tr(e^{-\beta Q})$$ (68)

and *Q* is the quasiparticle operator

$$Q=\frac{1}{2}\sum_{ij}\{h_{ij}(c_{i}^{†}c_{j}+c_{j}c_{i}^{†})+(\Delta_{ij}c_{i}^{†}c_{j}^{†}+\Delta_{ij}^{*}c_{j}c_{i})\}$$ (69)

whose matrix elements *h_ij_* and *∆_ij_* (defined as in Eqs. (24)–(25)) will be considered as variational parameters. In applying the variational principle, we seek to minimize the thermodynamic potential

ϕ(D)=E−μN−TS (70)

where *E* = *Tr* (*DĤ*) and $N=Tr(D\hat{N})$ are respectively the system’s average energy and particle number (*Ĥ* has been defined in Eq. (13) and $\hat{N}=\sum_{i}a_{i}^{†}a_{i}$ is the total number operator), *T* is the temperature and *S* the entropy of the system.

The functional we shall minimize is thus

$$\phi =-\frac{1}{\beta }\ln(Z_{0})-Tr(DQ)+\hat{E}$$ (71)

which represents an upper limit to the actual partition function *Z*. Here $Ê=Tr(D{\hat{H}}_{\mu })$ is the expectation value of the hamiltonian ${\hat{H}}_{\mu }=\hat{H}-\mu \hat{N}$.

We note that the single-particle (***ρ***) and pair density matrices (***κ***) are given in the statistical density matrix representation of a system by

$$\rho_{ij}=Tr[D(c_{j}^{†}c_{i})]$$ (72)

$$\kappa_{ij}=Tr[D(c_{j}c_{i})]$$ (73)

which can be summarized into

$$Tr(DQ)=\frac{1}{2}tr(HR)$$ (74)

where we discriminate between the trace operation in Fock space (*Tr*) and in the space of single-particle states (*tr*). Thus,

$$\delta [\ln(Z_{0})]=\frac{\beta }{2}tr(R\delta H)$$ (75)

Here *R* represents the generalized density matrix of a quasiparticle vacuum and *H* the quasiparticle hamiltonian, respectively defined in Eqs. (33) and (35).

We note that these are not the same as the ones defined in [11], but they are related by

$$R=\eta R_{0}\;H=H_{0}\;\eta$$ (76)

where the zero suffix has been used to represent the quantities defined in [11]. The matrix *η* appears here due to the nature of the boson commutation relations that must be satisfied by the transformed quasiparticle operators.

We also note that, for a particular state (which can be explicitly constructed from the particle vacuum), the generalized density matrix *R* obtains the canonical form

$$R=\left(\begin{array}{cc} 0 & 0\\ 0 & -11 \end{array}\right)$$ (77)

which satisfies the conditions

$$R^{†}=R\;R^{2}=-R$$ (78)

Using Wick’s theorem for ensemble averages, we can also express the energy *Ê* in terms of *R*, so that the minimization of the functional *ϕ*

$$\delta \phi =\frac{1}{2}tr(H\delta R)+\frac{\partial \hat{E}}{\partial R}\partial R=0$$ (79)

gives

$$tr\left\{\frac{\partial \hat{E}[R]}{\partial R}-\frac{1}{2}H\right\}\delta R=0$$ (80)

where we have used the notation [11]

$$tr\left[\left(\frac{\partial \hat{E}[R]}{\partial R}\right)\delta R\right]\equiv \sum_{ij}\frac{\partial \hat{E}[R]}{\partial R_{ji}}\delta R_{ji}$$ (81)

We hence deduce that *ϕ* is stationary with respect to arbitrary variations of *H* (or equivalently of *R* related to *H*) if^3^

$$\frac{\partial \hat{E}[R]}{\partial R_{ji}}=\frac{1}{2}H_{ij}$$ (82)

At steady state the energy functional *Ê* [*R*] remains constant, so that the second constraint of Eq. (78) can be taken into account by introducing a matrix of Lagrange multipliers *L* as

$$\delta \{\hat{E}[R]-tr[L(R^{2}+R)]\}=0.$$ (83)

Eliminating the Lagrange multipliers, we thus obtain the equation

[H,R]=0 (84)

which simply expresses Eqs. (60) and (61) in a more compact form. In order to obtain the full set of the steady state Hartree-Fock-Bogoliubov equations, we also need an equation for the vectors ***z***. This is obtained in [11] by consideration of the variation of the energy functional *Ê* [*R*] with respect to ***z*** and ***z***^*^. The resulting equation is identical to Eq. (66).
